# Pharmaceutical pollution of the world’s rivers

**DOI:** 10.1073/pnas.2113947119

**Published:** 2022-02-14

**Authors:** John L. Wilkinson, Alistair B. A. Boxall, Dana W. Kolpin, Kenneth M. Y. Leung, Racliffe W. S. Lai, Cristóbal Galbán-Malagón, Aiko D. Adell, Julie Mondon, Marc Metian, Robert A. Marchant, Alejandra Bouzas-Monroy, Aida Cuni-Sanchez, Anja Coors, Pedro Carriquiriborde, Macarena Rojo, Chris Gordon, Magdalena Cara, Monique Moermond, Thais Luarte, Vahagn Petrosyan, Yekaterina Perikhanyan, Clare S. Mahon, Christopher J. McGurk, Thilo Hofmann, Tapos Kormoker, Volga Iniguez, Jessica Guzman-Otazo, Jean L. Tavares, Francisco Gildasio De Figueiredo, Maria T. P. Razzolini, Victorien Dougnon, Gildas Gbaguidi, Oumar Traoré, Jules M. Blais, Linda E. Kimpe, Michelle Wong, Donald Wong, Romaric Ntchantcho, Jaime Pizarro, Guang-Guo Ying, Chang-Er Chen, Martha Páez, Jina Martínez-Lara, Jean-Paul Otamonga, John Poté, Suspense A. Ifo, Penelope Wilson, Silvia Echeverría-Sáenz, Nikolina Udikovic-Kolic, Milena Milakovic, Despo Fatta-Kassinos, Lida Ioannou-Ttofa, Vladimíra Belušová, Jan Vymazal, María Cárdenas-Bustamante, Bayable A. Kassa, Jeanne Garric, Arnaud Chaumot, Peter Gibba, Ilia Kunchulia, Sven Seidensticker, Gerasimos Lyberatos, Halldór P. Halldórsson, Molly Melling, Thatikonda Shashidhar, Manisha Lamba, Anindrya Nastiti, Adee Supriatin, Nima Pourang, Ali Abedini, Omar Abdullah, Salem S. Gharbia, Francesco Pilla, Benny Chefetz, Tom Topaz, Koffi Marcellin Yao, Bakhyt Aubakirova, Raikhan Beisenova, Lydia Olaka, Jemimah K. Mulu, Peter Chatanga, Victor Ntuli, Nathaniel T. Blama, Sheck Sherif, Ahmad Zaharin Aris, Ley Juen Looi, Mahamoudane Niang, Seydou T. Traore, Rik Oldenkamp, Olatayo Ogunbanwo, Muhammad Ashfaq, Muhammad Iqbal, Ziad Abdeen, Aaron O’Dea, Jorge Manuel Morales-Saldaña, María Custodio, Heidi de la Cruz, Ian Navarrete, Fabio Carvalho, Alhaji Brima Gogra, Bashiru M. Koroma, Vesna Cerkvenik-Flajs, Mitja Gombač, Melusi Thwala, Kyungho Choi, Habyeong Kang, John L. Celestino Ladu, Andreu Rico, Priyanie Amerasinghe, Anna Sobek, Gisela Horlitz, Armin K. Zenker, Alex C. King, Jheng-Jie Jiang, Rebecca Kariuki, Madaka Tumbo, Ulas Tezel, Turgut T. Onay, Julius B. Lejju, Yuliya Vystavna, Yuriy Vergeles, Horacio Heinzen, Andrés Pérez-Parada, Douglas B. Sims, Maritza Figy, David Good, Charles Teta

**Affiliations:** ^a^Department of Environment and Geography, University of York, York YO10 5DD, United Kingdom;; ^b^US Geological Survey, Central Midwest Water Science Center, Iowa City, IA 52240;; ^c^State Key Laboratory of Marine Pollution, Department of Chemistry, City University of Hong Kong, Hong Kong, China;; ^d^Center for Genomics, Ecology & Environment, Universidad Mayor, 8580745 Santiago, Chile;; ^e^Escuela de Medicina Veterinaria, Facultad de Ciencias de la Vida, Universidad Andres Bello, 8370035 Santiago, Chile;; ^f^Life and Environmental Sciences, Deakin University, Warrnambool 3280 VIC, Australia;; ^g^Environment Laboratories, International Atomic Energy Agency, 98000 Monaco, Principality of Monaco;; ^h^ECT Oekotoxikologie GmbH, 65439 Flörsheim am Main, Germany;; ^i^Centro de Investigaciones del Medioambiente, Facultad de Ciencias Exactas, Universidad Nacional de la Plata, Consejo Nacional de Investigaciones Científicas y Técnicas de Argentina, CP 1900 La Plata Buenos Aires, Argentina;; ^j^Institute for Environment and Sanitation Studies, University of Ghana, Accra LG 1181, Ghana;; ^k^Plant Protection Department, Agricultural University of Tirana, Tirana 1000, Albania;; ^l^School of Public Health, Imperial College London, London SW7 2AZ, United Kingdom;; ^m^Doctorado en Medicina de la Conservación, Facultad Ciencias de la Vida, Universidad Andres Bello, 7550196 Santiago, Chile;; ^n^Faculty of Chemistry, Center for Ecological Safety, Yerevan State University, 0025 Yerevan, Armenia;; ^o^School of Chemistry, University of Sydney, Sydney 2006 NSW, Australia;; ^p^Department of Environmental Geosciences, University of Vienna, 1010 Vienna, Austria;; ^q^Department of Emergency Management, Patuakhali Science and Technology University, 8602 Patuakhali, Bangladesh;; ^r^Molecular Biology and Biotechnology Institute, Universidad Mayor de San Andres, 6042 La Paz, Bolivia;; ^s^Department of Microbiology, Tumor and Cell Biology, Karolinska Institute, Stockholm 171 77, Sweden;; ^t^Instituto Federal De Educacao, Ciencia e Tecnologia do Rio Grande do Norte, 1692 Natal, Brazil;; ^u^Center for Research in Environmental Risk Assessment, School of Public Health of University of Sao Paulo, 01246-904 Sao Paulo, Brazil;; ^v^Research Unit in Applied Microbiology and Pharmacology of Natural Substances, Research Laboratory in Applied Biology, Polytechnic School of Abomey-Calavi, University of Abomey-Calavi, BP 526 Abomey Calavi, Benin;; ^w^Department of Zoology, Faculty of Science and Technology, University of Abomey-Calavi, BP 526 Abomey Calavi, Benin;; ^x^Sciences Appliquées et Technologies, Université de Dédougou, Ouagadougou BP 176, Burkina Faso;; ^y^Department of Biology, University of Ottawa, Ottawa, ON K1N 6N5, Canada;; ^z^Global Monitoring of Pharmaceutical Consortium, York YO10 5NG, United Kingdom;; ^aa^Centre de Recherches Hydrologiques, l'Institut de Recherches Géologiques et Minières, BP 4110 Yaounde, Cameroon;; ^bb^Departamento de Ingeniería Geográfica, Universidad de Santiago de Chile, 9170022 Santiago, Chile;; ^cc^Environmental Research Institute, School of Environment, Guangdong Provincial Key Laboratory of Chemical Pollution and Environmental Safety & MOE Key Laboratory of Theoretical Chemistry of Environment, South China Normal University, Guangzhou 510006, P. R. China;; ^dd^Department of Chemistry, Universidad del Valle, Cali 25360, Colombia;; ^ee^National Pedagogical University of Kinshasa, Kinshasa 8815, Democratic Republic of Congo;; ^ff^Faculty of Sciences, Department F.-A. Forel for Environmental and Aquatic Sciences, University of Geneva, Geneva 1205, Switzerland;; ^gg^Ecole Normale Supérieure, Departement des Sciences et Vie de la Terre, Université Marien Ngouabi, BP 69 Brazzaville, Republic of the Congo;; ^hh^Department of Geography, Geology, and the Environment, Kingston University London, KT2 7LB Kingston, United Kingdom;; ^ii^Central American Institute for Studies on Toxic Substances, Universidad Nacional, 40101 Heredia, Costa Rica;; ^jj^Division for Marine and Environmental Research, Rudjer Boskovic Institute, 10000 Zagreb, Croatia;; ^kk^Nireas International Water Research Center, Department of Civil and Environmental Engineering, University of Cyprus, Nocosia 1678, Cyprus;; ^ll^Faculty of Environmental Sciences, Czech University of Life Sciences Prague, 165 00 Prague, Czech Republic;; ^mm^Institute of Biotechnology, Addis Ababa University, Addis Ababa 1176, Ethiopia;; ^nn^Laboratory of Ecotoxicology, lnstitut National de Recherche pour l’Agriculture, l’Alimentation et l’Environnement, 69100 Villeurbanne, France;; ^oo^Department of Water Resources, Ministry of Fisheries & Water Resources, Banjul, The Gambia;; ^pp^Faculty of Agricultural Sciences and Biosystems Engineering, Georgian Technical University, 380075 Tbilisi, Georgia;; ^qq^Center for Applied Geoscience, University of Tübingen, Tübingen 72074, Germany;; ^rr^School of Chemical Engineering, National Technical University of Athens, 10682 Athens, Greece;; ^ss^Research Centre in Sudurnes, University of Iceland, Reykjavík 600169, Iceland;; ^tt^Department of Civil Engineering, Indian Institute of Technology Hyderabad, Hyderabad 502285, India;; ^uu^Department of Biochemical Engineering and Biotechnology, Indian Institute of Technology Delhi, 110016 New Delhi, India;; ^vv^Faculty of Civil and Environmental Engineering, Environmental Management Technology Research Group, 40132 Bandung, Indonesia;; ^ww^Education and Extension Organization, Iranian Fisheries Science Research Institute, Agricultural Research, Tehran 1588733111, Iran;; ^xx^Department of Planning and Environmental Policy, University College Dublin, Dublin D14 E099, Ireland;; ^yy^Spatial Dynamics Lab, University College Dublin, Dublin 4, Ireland;; ^zz^Department of Soil and Water Sciences, Faculty of Agriculture, Food and Environment, Hebrew University of Jerusalem, 9190501 Jerusalem, Israel;; ^aaa^Centre de Recherches Oceanologiques, 7XP 56V Abidjan, Cote d’Ivoire;; ^bbb^School of Engineering and Digital Sciences, Nazarbayev University, Nur-Sultan 010000, Kazakhstan;; ^ccc^L. N. Gumilyov Eurasian National University, 010000 Nur-Sultan, Kazakhstan;; ^ddd^Department of Geology, University of Nairobi, Nairobi 00100, Kenya;; ^eee^Department of Biology, National of University of Lesotho, Maseru 180, Lesotho;; ^fff^Environmental Protection Agency of Liberia, 1000 Monrovia, Liberia;; ^ggg^Department of Environment, International Institute of Aquaculture and Aquatic Sciences, Universiti Putra Malaysia, 43400 Seri Kembangan, Malaysia;; ^hhh^Centre d’Expertise et de Recherche en Télémédecine et E-Santé, Centre Hospitalier Mère – Enfant, Le Luxembourg, Hamdallaye, Bamako, Mali;; ^iii^Department of Global Health-Amsterdam Institute for Global Health and Development, Amsterdam Universty Medical Center, University of Amsterdam, 1012 WX Amsterdam, The Netherlands;; ^jjj^Department of Fisheries Technology, Ecotoxicology Research Laboratory, Lagos State Polytechnic, 100001 Ikorodu, Nigeria;; ^kkk^Department of Chemistry, University of Gujrat, Gujrat 50700, Pakistan;; ^lll^Al-Quds Nutrition and Health Research Institute, Al-Quds University, Abu Dies, West Bank, Palestine;; ^mmm^Smithsonian Tropical Research Institute, Panama City 0843-03092, Republic of Panama;; ^nnn^Facultad de Medicina Humana, Universidad Nacional del Centro del Peru, 12004 Huancayo, Peru;; ^ooo^Department of Environmental Science, Southern Leyte State University-Hinunangan Campus, 6608 Hinunangan, Philippines;; ^ppp^Lancaster Environment Centre, Lancaster University, Lancaster LA1 4YQ, United Kingdom;; ^qqq^Department of Chemistry, School of Environmental Sciences, Njala University, Bo, Sierra Leone;; ^rrr^Veterinary Faculty, University of Ljubljana, SI 1000 Ljubljana, Slovenia;; ^sss^Water Centre, Council for Scientific and Industrial Research, 0184 Pretoria, South Africa;; ^ttt^Seoul National University, 599 Seoul, South Korea;; ^uuu^College of Natural Resources and Environmental Studies, University of Juba, Juba, South Sudan;; ^vvv^IMDEA Water Institute, Science and Technology Campus, University of Alcalá, 28801 Alcalá de Henares, Spain;; ^www^International Water Management Institute, Colombo 10120, Sri Lanka;; ^xxx^Department of Environmental Science, Stockholm University, 114 19 Stockholm, Sweden;; ^yyy^Institute for Ecopreneurship, School of Life Sciences, University of Applied Sciences and Arts Northwestern Switzerland, 4132 Muttenz, Switzerland;; ^zzz^Department of Environmental Engineering, Chung Yuan Christian University, 320 Taoyuan, Taiwan;; ^aaaa^Institute of Resources Assessment, University of Dar es Salaam, Dar es Salaam, Tanzania;; ^bbbb^Institute of Environmental Sciences, Bogazici University, 34342 Istanbul, Turkey;; ^cccc^Faculty of Science, Mbarara University of Science & Technology, 9MM5 6GF Mbarara, Uganda;; ^dddd^Biology Centre of the Czech Academy of Sciences, Institute of Hydrobiology, Ceske 37005 Budejovice, Czech Republic;; ^eeee^Department of the Environment, O. M. Beketov National University of Urban Economy in Kharkiv, 61002 Kharkiv, Ukraine;; ^ffff^Faculty of Chemistry, Universidad de la República, 11200 Montevideo, Uruguay;; ^gggg^Departamento de Desarrollo Tecnológico, Centro Universitario Regional del Este, Universidad de la República, CP 27000, Rocha, Uruguay;; ^hhhh^School of Science & Mathematics, College of Southern Nevada, Henderson, NV 89002;; ^iiii^Department of Molecular and Cellular Biology, University of Guelph, Guelph, ON N1G 2W1, Canada;; ^jjjj^Future Water Research Institute, Faculty of Engineering & Built Environment, University of Cape Town, 7700 Cape Town, South Africa

**Keywords:** pharmaceuticals, aquatic contamination, antimicrobials, global pollution, wastewater

## Abstract

Environmental exposure to active pharmaceutical ingredients (APIs) can have negative effects on the health of ecosystems and humans. While numerous studies have monitored APIs in rivers, these employ different analytical methods, measure different APIs, and have ignored many of the countries of the world. This makes it difficult to quantify the scale of the problem from a global perspective. Furthermore, comparison of the existing data, generated for different studies/regions/continents, is challenging due to the vast differences between the analytical methodologies employed. Here, we present a global-scale study of API pollution in 258 of the world’s rivers, representing the environmental influence of 471.4 million people across 137 geographic regions. Samples were obtained from 1,052 locations in 104 countries (representing all continents and 36 countries not previously studied for API contamination) and analyzed for 61 APIs. Highest cumulative API concentrations were observed in sub-Saharan Africa, south Asia, and South America. The most contaminated sites were in low- to middle-income countries and were associated with areas with poor wastewater and waste management infrastructure and pharmaceutical manufacturing. The most frequently detected APIs were carbamazepine, metformin, and caffeine (a compound also arising from lifestyle use), which were detected at over half of the sites monitored. Concentrations of at least one API at 25.7% of the sampling sites were greater than concentrations considered safe for aquatic organisms, or which are of concern in terms of selection for antimicrobial resistance. Therefore, pharmaceutical pollution poses a global threat to environmental and human health, as well as to delivery of the United Nations Sustainable Development Goals.

## Significance

Despite growing evidence of the deleterious effects on ecological and human health, little is known regarding the global occurrence of pharmaceuticals in rivers. Studies assessing their occurrence are available for 75 of 196 countries, with most research conducted in North America and Western Europe. This leaves large geographical regions relatively unstudied. Here, we present the findings of a global reconnaissance of pharmaceutical pollution in rivers. The study monitored 1,052 sampling sites along 258 rivers in 104 countries of all continents, thus representing the pharmaceutical fingerprint of 471.4 million people. We show that the presence of these contaminants in surface water poses a threat to environmental and/or human health in more than a quarter of the studied locations globally.

Active pharmaceutical ingredients (APIs) are emitted to the natural environment during their manufacture, use, and disposal. There is evidence that environmental exposure to APIs has deleterious effects on the health of ecosystems and humans (e.g., by selecting for antibiotic resistant bacteria, feminizing fish, and increasing the susceptibility of fish to predation) (1–4). To fully understand the likely effects of these pharmaceutical exposures, it is essential to understand the concentrations that occur in riverine environments.

While a large body of data are available on the concentrations of many APIs in surface waters (5), substantial gaps exist in our knowledge of such exposures globally (6). A recent review (7) showed that while extensive datasets are available (e.g., refs. 5 and 8) on concentrations of APIs in the United States, many European countries, and in China, we simply have no data for most countries of the world (121 of the 196 countries). For countries with data, information is typically only available for a small number of APIs with studies rarely monitoring more than 20 contaminants in a single method (7, 9). Comparison of these existing data are significantly hindered by the fact that many different analytical techniques and sample-collection methods have been used over a wide time period. This makes it challenging to establish the scale of the problem globally, meaning that research and management efforts cannot be focused on pharmaceuticals and regions of greatest risk. By focusing on countries in Europe and North America, we are likely only considering the “tip of the iceberg,” as concentrations of some APIs are likely to be orders-of-magnitude greater in unstudied regions that tend to have limited regulation, poorer treatment infrastructures, and higher disease prevalence (10).

Here, we present a truly global study of pharmaceutical occurrence in the rivers of >50% of the world’s countries (*n* = 1,052 sites). We present a unique, high-quality and comparable dataset on the concentrations of 61 APIs and selected compounds used in medicine and as lifestyle consumables (caffeine, nicotine). The targeted compounds were selected based on previous prioritization exercises and were expected to occur in the environment and to be of potential environmental concern (11, 12). The study employed one sensitive (Dataset S1) and internationally validated sampling and analytical method used in one research laboratory (13), enabling a true comparison of pharmaceutical exposure data on a global scale.

## Results and Discussion

### Global Reach.

**Fig. 1.**
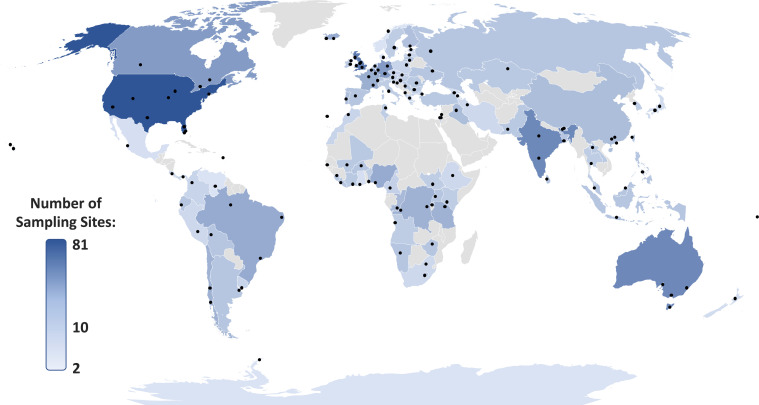
Locations of studied rivers/catchments (*n* = 137) for our global study (Dataset S2). Points indicate groups of sampling sites across respective river catchments and countries are shaded based upon the total number of sampling sites.

**Fig. 2.**
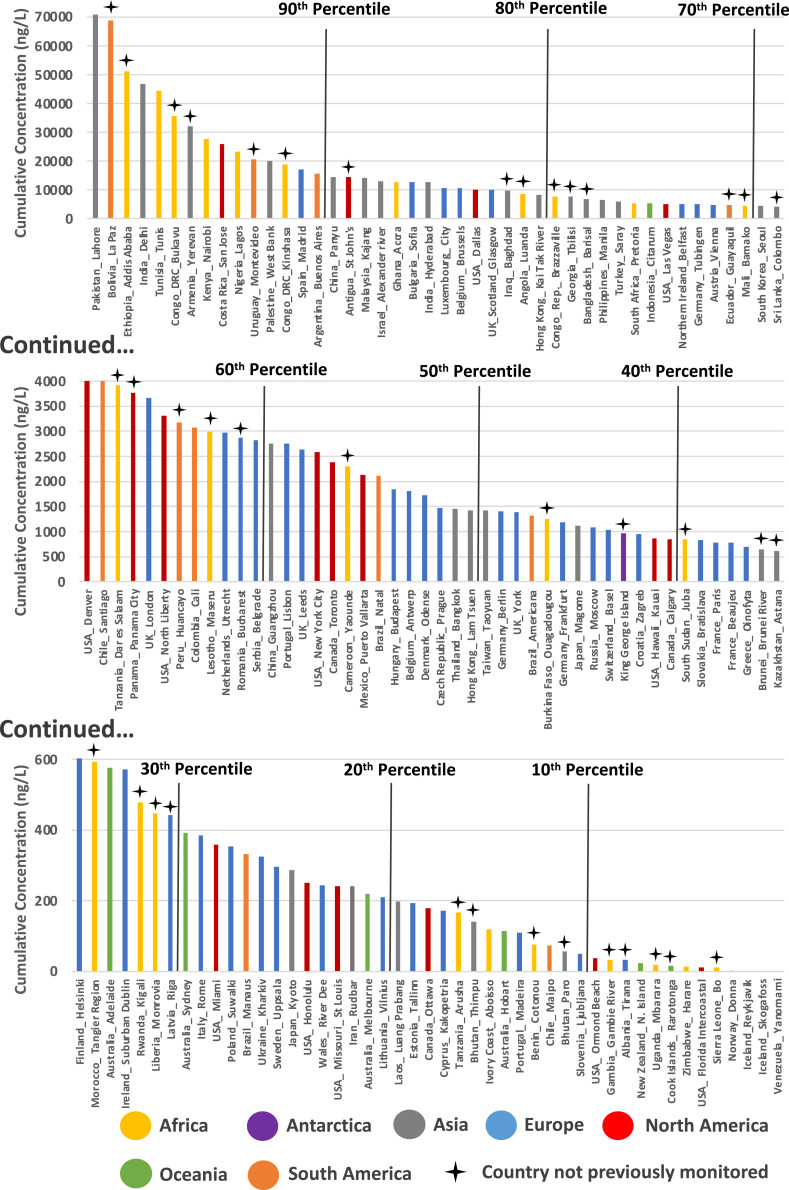
Cumulative API concentrations quantified across 137 studied river catchments (Dataset S6) organized by descending cumulative concentration (ng/L). Percentiles are marked by black lines and countries not previously monitored by crosses above the plot. The cumulative concentrations reported here are calculated as the average of the sum concentration of all quantifiable API residues at each sampling site within respective river catchments.

Surface water samples were collected in duplicate once from 1,052 sampling sites during 137 sampling campaigns covering 104 countries across all continents (Fig. 1 and Dataset S2) and analyzed for 61 APIs, resulting in 128,344 data points. A sampling campaign comprised the collection of water samples at a number of sampling sites along a river or rivers flowing within a city, a town, or local area. The number of sampling sites within a campaign ranged from 2 (Donna, Norway; Kyoto, Japan; and Antarctic Great Wall Station, Antarctica) to 18 (Denver, CO), with most campaigns including 5 to 11 sites (median number of sites = 8). The sampling included 24 countries in Africa (227 sampling sites), Antarctica (2 sampling sites), 24 in Asia (234 sampling sites), 37 in Europe (344 sampling sites), 6 in North America (118 sampling sites), 3 in Oceania (35 sampling sites), and 9 in South America (92 sampling sites). Of these and based on the UBA database of pharmaceuticals in the environment (5), 36 countries had never been monitored previously for APIs (Fig. 2 and Dataset S3).

The study included sampling sites with a broad suite of anthropogenic influences, spanning from a Yanomani Village (an Indigenous people of the Amazon Region) in Venezuela, where modern medicines are not used, to some of the most populated cities on the planet (e.g., Delhi, Seoul, New York, Kinshasa, and London). Areas of political instability were also included in the study (e.g., Baghdad in Iraq, Nablus in the Palestinian West Bank, and Yaoundé in Cameroon). The climates where samples were obtained varied from high altitude (>4,000 m) alpine tundra (e.g., Colorado, United States) and polar regions (e.g., Antarctica) to desert (e.g., Tunisia), and included all major climatic zones.

Sampling campaigns were performed in all European Union member states except Malta, which was not included due to the country’s lack of rivers. A total of 67 river catchments were monitored across the European Union. The most extensively studied country in this work was the United States. Here, 81 sampling sites were monitored (Dataset S2) along 29 rivers across 8 states (Colorado, Florida, Hawaii, Iowa, Missouri, Nevada, New York, and Texas).

### Cumulative Pharmaceutical Concentrations.

Cumulative pharmaceutical concentrations were calculated at each sampling site as the sum of all API residues quantified at that specific location. The mean of the cumulative concentrations was then determined across all the sites within a sampling campaign. With the exception of the campaigns in Iceland (17 sampling sites in total) and the Yanomami Village in Venezuela (3 sampling sites), at least one API was detected in all of our study campaigns. The highest mean cumulative concentration was observed in Lahore, Pakistan at 70.8 µg/L, with one sampling site reaching a maximum cumulative concentration of 189 µg/L (Fig. 2 and Dataset S4). This was followed by La Paz, Bolivia (68.9 µg/L mean, 297 µg/L maximum) and Addis Ababa, Ethiopia (51.3 µg/L mean, 74.2 µg/L maximum). The most polluted sampling site was located in the Rio Seke (La Paz, Bolivia) and had a cumulative API concentration of 297 µg/L (Dataset S4). This sampling site was associated with both untreated sewage discharge and disposal of rubbish along the bank of the river (Dataset S2).

The most contaminated samples were predominately from sampling campaigns in African (e.g., Ethiopia > Tunisia > Democratic Republic of Congo > Kenya > Nigeria) and Asian (Pakistan > India > Armenia > Palestine > China) countries. The most polluted North American samples were obtained from a campaign in San Jose, Costa Rica (mean 25.8 µg/L, maximum 63.1 µg/L: rank 9 of 137). The most polluted European samples were from a campaign in Madrid, Spain (mean 17.1 µg/L, maximum 59.5 µg/L: rank 14 of 137) and the most polluted Oceania samples were from a campaign in Adelaide, Australia (mean 0.577 µg/L, maximum 0.75 µg/L: rank 93 of 137) (Fig. 2 and Dataset S4).

Many of the most heavily contaminated samples were obtained from campaigns in low- to middle-income countries that had received limited or no previous monitoring of APIs in the aquatic environment. For example, of countries within the top 10th percentile for cumulative API concentrations across respective catchments, only three prior publications are available for Nigeria, two for Tunisia, one for Costa Rica and Palestine, and none for Armenia, the Democratic Republic of the Congo, Ethiopia, and Bolivia (5). Where previous research has been most intense (e.g., in the United States and Germany, with >300 previous publications in each country), total concentrations were generally substantially lower compared to lesser-studied regions (Fig. 2 and *SI Appendix*, Fig. S1) indicating that previous research effort has primarily focused on areas where lower risks to ecosystem and human health are likely.

On-the-ground observations made by sampling teams during sample collection (Dataset S2) revealed that the highest API concentrations were observed at: 1) sampling sites receiving inputs from pharmaceutical manufacturing (e.g., Barisal, Bangladesh, and Lagos, Nigeria), 2) sites receiving discharge of untreated sewage (e.g., Tunis, Tunisia, and Nablus, Palestine), 3) locations in particularly arid climates (e.g., Madrid, Spain), and 4) sites receiving sewage exhauster truck emissions and waste dumping (e.g., Nairobi, Kenya and Accra, Ghana). Sites with lowest API concentrations were typically characterized as having: 1) limited anthropogenic influence (e.g., alpine regions of the Rocky Mountains and the Ellidaár River in Iceland), 2) limited use of modern medicine (e.g., a remote Yanonamei Village in Venezuela), 3) sophisticated wastewater treatment infrastructure (e.g., Basel, Switzerland), and 4) high riverine flows with a large dilutional component (e.g., the Amazon River downstream from Manaus, Brazil, the Mississippi River in St. Louis, United States, and the Mekong River in Luang Prabang, Laos).

### Pharmaceutical Detection Frequencies and Concentrations.

Of the 61 targeted APIs (Dataset S1), 53 were detected in at least one sampling site (Dataset S3). On a continental basis, 4 APIs were detected in sampling sites in Antarctica, 21 in Oceania, 35 in South America, 39 in North America, 41 in Africa, 45 in Europe, and 48 in Asia (Dataset S3), with 4 APIs detected on all continents. Of the four APIs detected across all continents, all were considered either lifestyle compounds or over-the-counter APIs: caffeine (stimulant and lifestyle compound), nicotine (stimulant and lifestyle compound), acetaminophen/paracetamol (analgesic), and cotinine (metabolite of a stimulant and lifestyle compound). An additional 14 APIs were detected in all continents except Antarctica: atenolol (β-blocker), carbamazepine (antiepileptic), cetirizine (antihistamine), citalopram (antidepressant), desvenlafaxine (antidepressant), fexofenadine (antihistamine), gabapentin (anticonvulsant), lidocaine (anesthetic), metformin (antihyperglycemic), naproxen (anti-inflammatory), sitagliptin (antihyperglycemic), temazepam (benzodiazepine for insomnia treatment), trimethoprim (antimicrobial), and venlafaxine (antidepressant).

Cloxacillin (antimicrobial), diphenhydramine (antihistamine), miconazole (antimicrobial), norfluoxetine (antidepressant), oxazepam (benzodiazepine), oxytetracycline (antimicrobial), raloxifene (osteoporosis treatment), and sertraline (antidepressant) were not detected in any water sample. The lack of detection of cloxacillin is likely due to the hydrolytic instability of β-lactams in the natural environment (14). The lack of detection of oxytetracycline, miconazole, and sertraline may be explained by the propensity of these APIs to partition from the aqueous phase to environmental solids (15, 16). The lack of detection for norfluoxetine may be explained by the relatively high limits of quantification for this API compared to others in our analytical method (13).

**Fig. 3.**
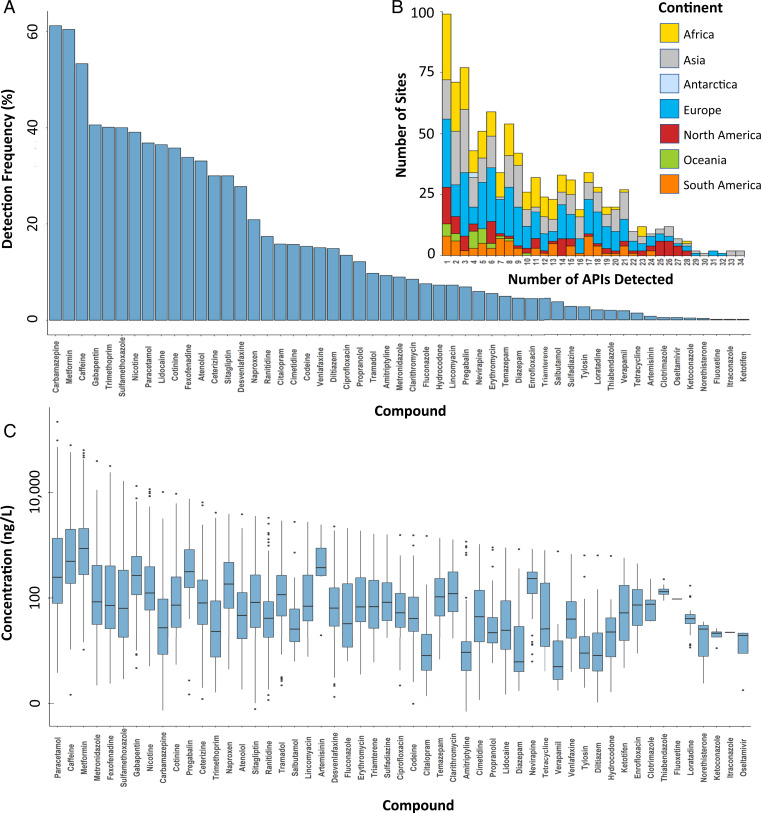
(*A*) Detection frequencies (Dataset S5) and (*B*) number of APIs detected at sampling sites in the global monitoring study (Dataset S4), excluding sites without the detection of any API, and (*C*) box-and-whisker plots of concentrations (ng/L) of individual APIs (Dataset S4), indicating the mean, minimum, maximum, and upper and lower quartile concentrations for each API globally.

For the detected APIs, overall detection frequencies ranged from 0.1% for fluoxetine (antidepressant), itraconazole (antifungal), and ketotifen (antihistamine), to 62% for carbamazepine (Fig. 3*A*) within respective river catchments. Metformin and caffeine were also detected at over 50% of all the sampling sites worldwide (Fig. 3*A* and Dataset S5).

While detection frequencies of some APIs (e.g., carbamazepine, metformin, caffeine, nicotine, acetaminophen/paracetamol, and cotinine) were similar across continents, others revealed clear geographical differences (Datasets S3 and S5). Overall, API detection frequencies for Oceania were generally lower than in Europe, North America, and South America (Dataset S3). Detection frequencies for gabapentin, fexofenadine, cetirizine, sitagliptin, ranitidine, citalopram, and enrofloxacin (antimicrobial) in Africa were lower than in Asia, Europe, North America, and South America, while detection frequencies of cimetidine were lower in Europe and North America than in Africa and Asia (Datasets S3 and S5). Artemisinin (antimalarial) and clotrimazole (antifungal) were only detected in Africa, while oseltamivir (antiviral) and ketoconazole (antifungal) were only detected in Asia.

The contaminants with the highest concentrations were paracetamol, caffeine, metformin, fexofenadine, sulfamethoxazole (antimicrobial), metronidazole (antimicrobial), and gabapentin (Fig. 3*C* and Dataset S3). The highest concentration for any API was 227 µg/L for paracetamol at a sampling site on the Rio Seke (a small and heavily polluted river) in La Paz, Bolivia, where the local sampling team noted evidence of septic tank exhauster and rubbish dumping upstream of the sampling site (Dataset S2).

Clear global geographical patterns emerged in the API concentrations of key therapeutic classes (*SI Appendix*, Fig. S1). While total concentrations of some APIs (e.g., β-blockers and antihistamines) showed a relatively limited two to three orders-of-magnitude global range (i.e., the range of concentrations observed worldwide) and one to two orders-of-magnitude intercontinental variation in concentrations (i.e., the difference in concentrations between continents), others were substantially more varied (*SI Appendix*, Fig. S1 and Dataset S7). The largest global concentration range was observed for APIs from the analgesic, antibiotic, and anticonvulsant classes (approximately four to five orders-of-magnitude each).

Likely contributing to this large range in API concentrations are the relative affordability and differences in regulatory oversight of the accessibility of these medicines (17–19). Regions with less regulated access to medicines (e.g., regions where antibiotics are available over the counter) generally revealed greater variability and range of API concentrations (Datasets S4 and S7). This trend was most notable for antibiotic medicines in African countries, which showed both the highest variability (four orders-of-magnitude) and concentrations (threefold higher on average than the next closest continent) worldwide (*SI Appendix*, Fig. S1*B*). This may, in part, be driven by a general lack of enforceable regulatory oversight for proper antibiotic sales and use in human (20–23) and veterinary (24, 25) applications.

### The Socioeconomics of Pharmaceutical Pollution.

**Fig. 4.**
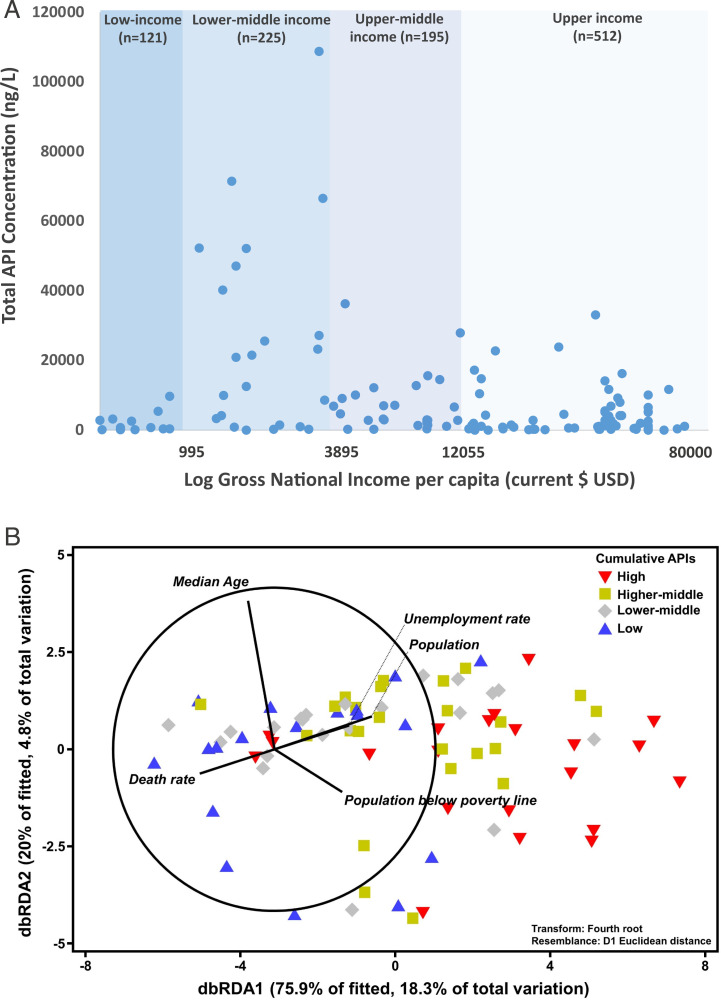
(*A*) Cumulative concentration of APIs (Dataset S6) observed across respective river catchments (signified by a blue dot, *n* = number of sampling sites) organized by World Bank GNI per capita (33) and (*B*) distance-based redundancy analysis (dbRDA) illustrating the best model of socioeconomic indicators to explain the measured concentration of different classes of pharmaceuticals in respective countries according to the distance-based linear model (DISTLM, AICc = 325.26, *r*^2^ = 0.241). Vector projections with center coordination at (−3, 0) were performed with multiple partial correlation. Length and direction of the vectors represent the strength and direction of the relationship. Data from each country were classified according to their cumulative active pharmaceutical ingredient concentration: that is, Low: first quartile (the lowest 25%); Lower-middle: second quartile (the next 25%); Higher-middle: third quartile (the next 25%); and High: fourth quartile (the top 25%). Raw data can be found in Dataset S9.

Recent modeling indicates that socioeconomic drivers may, in part, help explain the environmental distribution of APIs (26). In this study, concentrations of APIs were found to be highest in countries of lower-middle income (gross national income [GNI]-index: 995 to 3,895 USD$) than in countries of any other income-classification reported by the World Bank (Fig. 4*A* and Dataset S6). A statistically significant difference was observed between API concentrations of the different World Bank income classifications (one-way ANOVA, *F* = 14.2, *P* < 0.001) (Dataset S6), with a Tukey’s post hoc test indicating that this difference lies between that of the lower-middle income and all other categories of cumulative API concentrations (*P* < 0.001) (Dataset S6). Although speculative, this relationship may be explained as lower-middle income countries typically have low connectivity to wastewater infrastructure (27) while also tending to have improved access to larger numbers of medicines relative to low-income countries with lower healthcare expenditures (28–30). Hence, increasing access to medicines in lower-middle income countries relative to those of low-incomes, in conjunction with limited wastewater treatment infrastructure, likely leads to the highest concentrations of APIs in rivers globally. In contrast, while low-income countries will also have limited wastewater and waste management infrastructure, the access and affordability of medicines in these countries is also low, and hence so too are environmental API concentrations (28, 31). Upper-middle and high-income countries, while having access to medicines, typically have higher connectivity to wastewater treatment, more sophisticated waste management systems, and tighter regulation of medicinal use (29, 32), thus resulting in relatively lower environmental API concentrations.

Similarly, differences in the therapeutic compositions of API pollution were also observed based on the GNI-index of respective countries (Datasets S7 and S9) and, in particular, between those of lower-middle and high income indices (*SI Appendix*, Fig. S2 and Dataset S7). Comparing cumulative pharmaceutical concentrations in the low-to-middle income countries (*n* = 536 sampling sites) to that of countries with a high GNI-index (*n* = 512 sites) as defined by the World Bank (33), statistically significant differences were observed (one-way ANOVA followed by Tukey’s post hoc test; *F* = 13.4; *P* < 0.001) (Datasets S6 and S7). Of these, antihyperglycemic (*P* < 0.001) (Dataset S7) and antidepressant (*P* = 0.006) (Dataset S7) medicines made up a significantly smaller (Dataset S7) proportion of the cumulative API concentration in low-to-middle income countries than those observed in the high incomes (*SI Appendix*, Fig. S2). However, occurrence of analgesics and antibiotics were significantly more dominant (*P* < 0.001, respectively) (Dataset S7) in low-to-middle income countries, making up 29% and 15% of therapeutic composition of API concentrations detected, relative to 11% and 4% in high-income countries, respectively (*SI Appendix*, Fig. S2).

Complementing this finding, statistical associations were determined between API pollution and specific socioeconomic variables underpinning national economies and health via distance-based linear modeling. Here, pharmaceutical pollution was most positively associated with population, median age, local unemployment, and poverty rates and negatively associated with the death rate of a country (Fig. 4*B*) (Akaike Information Criterion [AICc] = 325.26, *P* = 0.025, cumulative *r*^2^ = 0.241). Among them, population is the most significant factor (Dataset S10). Multicollinearity results further confirmed the relationship between national API pollution and respective economies, health, and wastewater treatment facilities (Dataset S11). For example, the colinear socioeconomic indicators of the most significant factor, population, include disability-adjusted life years attributable to the environment (*r* = 0.95), real gross domestic product (*r* = 0.74), and the amount of produced, collected, and untreated municipal wastewater (*r* = 0.66 to 0.69). Although further work is needed, these global data reinforce the hypothesis that socioeconomics and human health are key predictors of environmental pollution from medicinal substances. Future work may use such indicators for prioritization of locations for both environmental monitoring (particularly where capacity is a limiting factor) and potential mitigation measures.

### Implications of Global Pharmaceutical Pollution for Ecological and Human Health.

As APIs are biologically active molecules, specifically designed to interact with biochemical pathways, of which many are conserved in both aquatic and terrestrial organisms, concerns have been raised over deleterious ecological implications of APIs in the aquatic environment. In Europe, for new APIs where environmental exposure is expected, ecotoxicological testing is required as part of the market authorization process (34). These tests explore the effects of APIs on the growth of cyanobacteria and green algae and the growth and reproduction of invertebrates and fish. Resulting data are then used to derive predicted no-effect concentrations (PNECs) for an API in the environment of interest. Recent papers have compiled PNECs for a range of APIs (35, 36). Data on the potency of APIs in humans alongside predictions on uptake into aquatic organisms have also been used to develop critical environmental concentrations (CECs) for APIs (37), the assumption being that if concentrations in the plasma of aquatic organisms reaches levels close to human plasma therapeutic concentrations, then effects are possible. These PNECs and CECs can be used to identify APIs that may be of concern in a particular system.

**Fig. 5.**
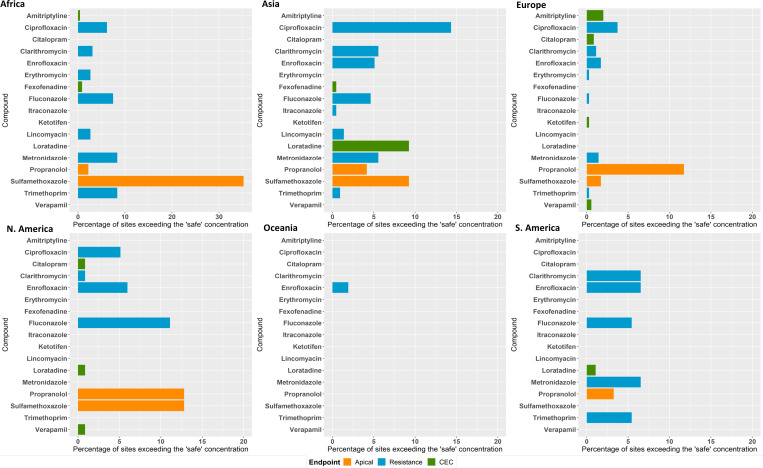
Percent of sites in the global monitoring study where concentrations exceeded: lowest PNECs (Dataset S12) derived from apical ecotoxicological endpoints for algae, fish, and daphnia (orange bars); CECs estimated based on human plasma therapeutic concentrations and uptake predictions for fish (green bars); and “safe” target concentrations for AMR selection (blue bars).

Comparison of available PNECs (35, 36) and CECs (37) for our study APIs (Dataset S12) with the corresponding exposure results (Dataset S4) show that, for most APIs, concentrations observed in rivers globally are lower than concentrations that could cause ecological effects. The exceptions were sulfamethoxazole (antimicrobial), propranolol (β-blocker), loratadine (antihistamine), amitriptyline (antidepressant), citalopram (antidepressant), fexofenadine (antihistamine), verapamil (Ca channel blocker), and ketotifen (antihistamine). Environmental concentrations exceeded PNEC values for at least one of the studied APIs at 270 of 1,052 study sites (25.7%). For sulfamethoxazole, 140 monitoring sites had concentrations above the PNEC (Fig. 5 and Dataset S12). Our data also clearly show that organisms in riverine systems are exposed to complex mixtures of APIs (Fig. 3*B*). The highest number of APIs detected at a single site was 34 at a location in the Kai Tak River in Hong Kong (Dataset S4). Ecological risks, therefore, could well be greater than predicted for the single APIs due to toxicological interactions of these mixtures (38).

For antimicrobial APIs, there is also concern that environmental exposures could select for antimicrobial resistance (AMR) in microorganisms and thus contribute to the global AMR crisis. A series of “safe” target concentrations (Dataset S12) were recently proposed for these APIs (39, 40) to protect against resistance selection. Concentrations of 9 of the 13 detected antimicrobials (Fig. 5) exceeded these safe concentrations for at least one sampling site, with ciprofloxacin exceeding the safe limit at 64 sites. The greatest exceedance of the safe target was observed for metronidazole at a sampling site in Barisal, Bangladesh, where the highest concentration of this antibiotic was more than 300 times higher than the safe target. On-the-ground observation made by the sampling team at this site noted the presence of wastewater disposal along the river and the close proximity of pharmaceutical manufacturing activities (Dataset S2).

### Toward 2030: The New Paradigm in Environmental Monitoring.

This study demonstrates how the use of a minimized-design sampling protocol with rapid and cost-effective analytical methodologies and a well-connected global community allows us to investigate API exposures and subsequent risks in rivers on a truly global scale. While this study focused on 61 priority APIs, the approach could be applied to other APIs and other classes of pollutants, such as personal care products, endocrine disrupting chemicals, pesticides, and metals. The integration of nontargeted analytical methods could also allow for the identification of unknown global pollutants.

In the future, our approach could also be expanded to other environmental media, such as sediments, soils, and biota. This would allow for the development of global-scale datasets on pollution, which will be invaluable for the successful delivery of the United Nations’ Sustainable Development Goals (41), particularly Goal 6.3 (to improve water quality via a reduction in pollution, elimination of dumping, and to minimize the release of hazardous chemical material and untreated wastewater into the aquatic environment).

As a consortium of 127 authors representing 86 institutions worldwide, we demonstrate that pollution of the world’s rivers by medicinal chemicals is a global problem that: 1) poses risk to both aquatic ecology and potential AMR selection and 2) may risk achievement of the United Nations Sustainable Development Goal 6.3 by 2030. As we move toward 2030, the new paradigm in environmental monitoring must involve a global, inclusive, and interconnected effort. Only through global collaboration will we be able to generate the monitoring data required to make informed decisions on mitigation approaches required to reduce the environmental impacts of chemicals.

## Materials and Methods

Identical water sampling kits (*SI Appendix*, Image S1) were sent to project collaborators which contained: 20 × 5 mL amber glass vials, 10× plastic disposable syringes, 10× glass microfiber GFX syringe filters (0.45-µm pore size), a 50-mL sampling bucket with 6-m nylon cord, and an ice pack. Project collaborators were asked to design a sampling campaign comprising 5 to 10 sampling sites along rivers flowing through a populated area (village, town, or city). Sampling sites within a campaign would typically include sites upstream, within, and downstream from the populated area and sites associated with points of interest, such as wastewater treatment discharges or waste disposal sites. Discussion with each project collaborator enabled a characterization of potential sources of pharmaceutical pollution affecting each river catchment (e.g., untreated sewage discharge, hospitals, wastewater treatment plants, septic systems, and pharmaceutical manufacturing facilities).

Water collection occurred at each site by lowering the sampling bucket (which was first rinsed three times with native water) into the water using the attached cord. An aliquot of water was then aspirated into a syringe after an initial rinsing with the native water. The syringe filter was then attached, primed, and the glass sample vial was rinsed with filtrate before 4 mL of filtered sample was discharged into it. Photographs, and where possible, environmental data including pH, electrical conductivity, total dissolved solids, and river flow were collected at each site (Dataset S2). Videos and a step-by-step guide (13) were provided to all collaborators detailing the required sample collection protocol to ensure consistency across all sampling campaigns. Each site was duplicate sampled one time and all sites within respective river catchments were sampled on the same day (Dataset S2).

Samples were kept frozen after collection until being sent (also frozen) via express air shipment to a single analytical center in the United Kingdom for analysis using a single analytical method (13). The duration of return shipment ranged from 0.5 to 4 d (mean 1.43 ± 0.8 d) and a separate investigation (13) showed no significant degradation of the target pharmaceuticals over this period. Simulated shipping events showed that the interior temperature of the shipment box remained below ambient temperature for at least 2 d (13). Upon delivery at the University of York, samples were kept at −20 °C until analysis.

Analysis occurred at the Centre of Excellence in Mass Spectrometry located at the University of York (United Kingdom) by high-pressure liquid chromatography-tandem mass spectrometry (HPLC-MS/MS). A fully validated method (13) adapted from US Geological Survey method No. 5-B10 (11), was used for the specific quantification of 61 APIs (Dataset S1). Briefly, limits of detection (Dataset S1), ranging from 0.5 ng/L (Diltiazem) to 139 ng/L (Norfluoxetine), were achieved by direct injection of 100 µL of the field-filtered sample (13). Positive electrospray ionization was used to generate two transition ions per target analyte and internal standard, one transition for quantification and the other for confirmation. Analysis occurred using a Thermo Endura triple quadrupole mass spectrometer operated in multiple reaction monitoring mode with a Phenomenex Zorbax Eclipse C18 Plus chromatography column. Mobile phase A was LCMS-grade water with 0.01 M formic acid and 0.01 M ammonium formate while mobile phase B was 100% methanol. The HPLC gradient started at 10% B, which increased to 40% at 5 min, 60% at 10 min, 100% at 15 min, where it remained until 23 min, then reduced to 10% at 23.1 min prior to a 10-min re-equilibration period. Quantification was achieved using a 15-point calibration curve, ranging from 1 to 8,000 ng/L via Thermo Scientific TraceFinder 4.1 General Quantitation software. A total of 30 deuterated internal standards were used at a concentration of 80 ng/L each and robust quality control measures were employed throughout sample collection and analysis (*SI Appendix*).

Statistical analysis (*SI Appendix*) was conducted using Microsoft Excel, SPSS and Primer with PERMANOVA+ (v7.0.17, Primer-e). Population and socioeconomic data were obtained from the World Bank open database (33). Hazard quotients for an assessment of potential ecotoxicity risk were generated by dividing the observed environmental concentrations (Dataset S4) by the lowest predicted no-effect concentration (35, 36, 39, 40) or critical environmental concentration (37) derived for each studied API in the literature (Dataset S12).

## Supplementary Material

Supplementary File

Supplementary File

Supplementary File

Supplementary File

Supplementary File

Supplementary File

Supplementary File

Supplementary File

Supplementary File

Supplementary File

Supplementary File

Supplementary File

## Data Availability

All data generated in this study are available in this article, the associated datasets (namely Dataset S4), and *SI Appendix*.
