# Supplementary material for: Pharmaceutical pollution of the world’s rivers
Source: Proc Natl Acad Sci U S A. 2022 Feb 14;119(8):e2113947119. doi: 10.1073/pnas.2113947119 (PMC8872717; doi:10.1073/pnas.2113947119)
Supplement: Supplementary File [file pnas.2113947119.sapp.pdf]

Supporting Online Material for:

Pharmaceutical Pollution of the World's Rivers

John L. Wilkinson[1]*, Alistair B.A. Boxall[1], Dana W. Kolpin[2], Kenneth M. Y. Leung[3], Racliffe W. S. Lai[3], Cristóbal Galbán-Malagón[4], Aiko D. Adell[5], Julie Mondon[6], Marc Metian[7], Robert Marchant[1], Alejandra Bouzas-Monroy[1], Aida Cuni-Sanchez[1], Anja Coors[8], Pedro Carriquiriborde[9], Macarena Rojo[9], Chris Gordon[10], Magdalena Cara[11], Monique Moermond[‡], Thais Luarte[12], Vahagn Petrosyan[13], Yekaterina Perikhanyan[13], Clare S. Mahon[14], Christopher J. McGurk[14], Thilo Hofmann[15], Tapos Kormoker[16], Volga Iniguez[17], Jessica Guzman-Otazo[18], Jean L. Tavares[19], Francisco Gildasio De Figueiredo[19], Maria T. P. Razzolini[20], Victorien Dougnon[21], Gildas Gbaguidi[22], Oumar Traoré[23], Jules M. Blais[24], Linda E. Kimpe[24], Michelle Wong[‡], Donald Wong[‡], Romaric Ntchantcho[25], Jaime Pizarro[26], Guang-Guo Ying[27], Chang-Er L. Chen[27], Martha Páez[28], Jina Martínez-Lara[28], Jean-Paul Otamonga[29], John Pote[30], Suspense A. Ifo[31], Penelope Wilson[32], Silvia Echeverría-Sáenz[33], Nikolina Udikovic-Kolic[34], Milena Milakovic[34], Despo Fatta-Kassinos[35], Lida Ioannou-Ttofa[35], Vladimíra Belušová[36], Jan Vymazal[36], María Cárdenas-Bustamante[1], , Bayable A. Kassa[37], Jeanne Garric[38], Arnaud Chaumot[38], Peter Gibba[39], Ilia Kunchulia[40], Sven Seidensticker[41], Gerasimos Lyberatos[42], Halldór P. Halldórsson[43], Molly Melling[1], Thatikonda Shashidhar[44], Manisha Lamba[45], Anindrya Nastiti[46], Adee Supriatin[46], Nima Pourang[47], Ali Abedini[47], Omar Abdullah[1], Salem S. Gharbia[48], Francesco Pilla[49], Benny Chefetz[50], Tom Topaz[50], Koffi Marcellin Yao[51], Bakhyt Aubakirova[52], Raikhan Beisenova[53], Lydia Olaka[54], Jemimah K. Mulu[54], Peter Chatanga[55], Victor Ntuli[55], Nathaniel T. Blama[56], Sheck Sherif[56], Ahmad Zaharin Aris[57], Ley Juen Looi[57], Mahamoudane Niang[58], Seydou T. Traore[58], Rik Oldenkamp[59], Olatayo Ogunbanwo[60], Muhammad Ashfaq[61], Muhammad Iqbal[61], Ziad Abdeen[62], Aaron O'Dea[63], Jorge Manuel Morales-Saldaña[63], María Custodio[64], Heidi de la Cruz[64], Ian Navarrete[65], Fabio Carvalho[66], Alhaji Brima Gogra[67], Bashiru Mohamed Koroma[67], Vesna Cerkvenik-Flajs[68], Mitja Gombač[68], Melusi Thwala[69], Kyungho Choi[70], Habyeong Kang[70], John L. Celestino Ladu[71], Andreu Rico[72], Priyanie Amerasinghe[73], Anna Sobek[74], Gisela Horlitz[74], Armin K. Zenker[75], Alex C. King[75], Jheng-Jie Jiang[76], Rebecca Kariuki[1], Madaka Tumbo[77], Ulas Tezel[78], Turgut T. Onay[78], Julius B. Lejju[79], Yuliya Vystavna[80], Yuriy Vergeles[81], Horacio Heinzen[82], Andrés Pérez-Parada[83], Douglas B Sims[84], Maritza Figy[‡], David Good[85] and Charles Teta[86]

[1]University of York, Environment and Geography Department, York, United Kingdom; [2]U.S. Geological Survey, Central Midwest Water Science Center, Iowa City, IA, United States of America; [3]State Key Laboratory of Marine Pollution and Department of Chemistry, City University of Hong Kong, Tat Chee Avenue, Kowloon, Hong Kong, China; [4]GEMA, Center for Genomics, Ecology & Environment, Universidad Mayor, Camino La Pirámide 5750, Huechuraba, Santiago, Chile; [5]Escuela de Medicina Veterinaria, Facultad de Ciencias de la Vida, Universidad Andres Bello, Republica 440, Santiago, Chile; [6]Deakin University, Life and Environmental Sciences, Warrnambool, Victoria, Australia; [7]International Atomic Energy Agency (IAEA), Environment Laboratories, Monaco, Principality of Monaco; [8]ECT Oekotoxikologie GmbH, Flörsheim/Main, Germany; [9]Centro de Investigaciones del Medioambiente (CIM), Facultad de Ciencias Exactas, Universidad Nacional de la Plata – CONICET, Boulevard 120 N1489 (1900) La Plata Buenos Aires, Argentina; [10]Institute for Environment and Sanitation Studies, University of Ghana, Accra, Ghana; [11]Agricultural University of Tirana, Tirana, Albania; [12]Doctorado en Medicina de la Conservación, Facultad Ciencias de la Vida, Universidad Andres Bello, Santiago, Chile; [13]Faculty of Chemistry, Center for Ecological Safety, Yerevan State University, Yerevan, Armenia; [14]University of Sydney, Sydney, Australia; [15]University of Vienna, Department of Environmental Geosciences, Vienna, Austria; [16]Department of Emergency Management, Patuakhali Science and Technology University, Dumki, Patuakhali, Bangladesh; [17]Molecular Biology and Biotechnology Institute, La Paz, Bolivia; [18]Karolinska Institute, Stockholm, Sweden; [19]Instituto Federal De Educacao, Ciencia e Tecnologia do Rio Grande do Norte, Brazil; [20]School of Public Health of University of Sao Paulo, Center for Research in Environmental Risk Assessment, Sao Paulo, Brazil; [21]Research Unit in Applied Microbiology and Pharmacology of natural substances, Research Laboratory in Applied Biology, Polytechnic School of Abomey-Calavi, University of Abomey-Calavi, Benin; [22]Department of Zoology, Faculty of Science and Technology, University of Abomey-Calavi, Abomey Calavi, Benin; [23]Université de Dédougou, Burkina Faso; [24]University of Ottawa, Ottawa, Canada; [25]Centre de Recherches Hydrologiques de l'Institut de Recherches Géologiques et Minières, Yaounde, Cameroon; [26]Departamento de Ingeniería Geográfica, Universidad de Santiago de Chile, Santiago, Chile; [27]Environmental Research Institute, School of Environment, Guangdong Provincial Key Laboratory of Chemical Pollution and Environmental Safety & MOE Key Laboratory of Theoretical Chemistry of Environment, South China Normal University, Guangzhou 510006, P.R. China; [28]Universidad del Valle, Cali, Colombia; [29]National Pedagogical University of Kinshasa, Kinshasa, Democratic Republic of Congo; [30]University of Geneva, Geneva, Switzerland; [31]Ecole Normale Supérieure, Departement des sciences et vie de la terre, Université Marien Ngouabi, Brazzaville, Republic of the Congo; [32]Department of Geography, Geology and the Environment, Kingston University London, Kingston, United Kingdom; [33]Central American Institute for Studies on Toxic Substances (IRET), Universidad Nacional (UNA), Heredia, Costa Rica; [34]Division for Marine and Environmental Research, Rudjer Boskovic Institute, Zagreb, Croatia; [35]Department of Civil and Environmental Engineering and Nireas-International Water Research Center, University of Cyprus; [36]Czech University of Life Sciences Prague, Faculty of Environmental Sciences, Prague, Czech Republic; [37]Institute of Biotechnology, Addis Ababa University, Addis Ababa, Ethiopia; [38]Institut national de recherche pour l'agriculture, l'alimentation et l'environnement, Laboratory of ecotoxicology, Villeurbanne, France; [39]Department of Water Resources, Banjul, The Gambia; [40]Faculty of Agricultural Sciences and Biosystems Engineering, Georgian Technical University, Tbilisi, Georgia; [41]University of Tübingen, Tübingen, Germany; [42]National Technical University of Athens, School of Chemical Engineering, Athens, Greece; [43]The University of Iceland´s Research Centre in Sudurnes, Iceland; [44]Indian Institute of Technology Hyderabad, Kandi, Sangareddy District 502285, Telangana, India Pin code: 502284; [45]Department of Biochemical Engineering and Biotechnology, Indian Institute of Technology Delhi; [46]Environmental Management Technology Research Group, Faculty of Civil and Environmental Engineering, Bandung, Indonesia; [47]Iranian Fisheries Science Research Institute (IFSRI), Agricultural Research, Education and Extension Organization (AREEO), Tehran, Iran; [48]IT Sligo, Sligo, Ireland; [49]Spatial

Dynamics Lab, University College Dublin, Dublin, Ireland; [50]Department of Soil and Water Sciences, Faculty of Agriculture, Food and Environment, Hebrew University of Jerusalem, Israel; [51]Centre de Recherches Oceanologiques, Abidjan, Cote d'Ivoire; [52]Nazarbayev University, Nur-Sultan, Kazakhstan; [53]L.N.Gumilyov Eurasian National University, Nur-Sultan, Kazakhstan; [54]Department of Geology, University of Nairobi, Nairobi, Kenya; [55]Department of Biology, National of University of Lesotho, Maseru, Lesotho; [56]Environmental Protection Agency of Liberia, Monrovia, Liberia; [57]Department of Environment, International Institute of Aquaculture and Aquatic Sciences (i-AQUAS), Universiti Putra Malaysia, Selangor, Malaysia; [58]Centre d'Expertise et de Recherche en Télémédecine et E-Santé, Bamako, Mali; [59]Department of Global Health-Amsterdam Institute for Global Health and Development, Amsterdam UMC, University of Amsterdam, Amsterdam, The Netherlands; [60]Department of Fisheries Technology, Ecotoxicology Research Laboratory, Lagos State Polytechnic, Ikorodu, Lagos State, Nigeria; [61]University of Gujrat, Gujrat, Pakistan; [62]Al-Quds Nutrition and Health Research Institute, Al-Quds University, Abu Dies, West Bank; [63]Smithsonian Tropical Research Institute, Balboa, Republic of Panama; [64]Facultad de Medicina Humana, Universidad Nacional del Centro del Peru, Huancayo, Peru; [65]Department of Environmental Science, Southern Leyte State University-Hinunangan Campus, Ambacon, Hinunangan, Southern Leyte, Philippines; [66]Lancaster Environment Centre, Lancaster University, Lancaster, LA1 4YQ, UK; [67]Department of Chemistry, School of Environmental Sciences, Njala University, Bo, Sierra Leone; [68]University of Ljubljana, Veterinary Faculty, Ljubljana, Slovenia; [69]Water Centre, Council for Scientific and Industrial Research, Pretoria, South Africa; [70]Seoul National University, Seoul, South Korea; [71]College of Natural Resources and Environmental Studies, University of Juba, Juba, South Sudan; [72]IMDEA Water Institute, Science and Technology Campus of the University of Alcalá, Alcalá de Henares, Spain; [73]International Water Management Institute, Colombo, Sri Lanka; [74]Department of Environmental Science, Stockholm University, Sweden; [75]Institute for Ecopreneurship, School of Life Sciences, University of Applied Sciences and Arts Northwestern Switzerland, Muttenz, Switzerland; [76]Department of Environmental Engineering, Chung Yuan Christian University, Taoyuan, Taiwan; [77]University of Dar es Salaam, Dar es Salaam, Tanzania; [78]Institute of Environmental Sciences, Bogazici University, Istanbul, Turkey; [79]Mbarara University of Science & Technology, Faculty of Science, Mbarara, Uganda; [80]Biology Centre of the Czech Academy of Sciences, Institute of Hydrobiology, Ceske Budejovice, Czech Republic; [81]O.M. Beketov National University of Urban Economy in Kharkiv, Department of the Environment, Kharkiv, Ukraine; [82]Faculty of Chemistry, Universidad de la República, Montevideo, Uruguay; [83]Departamento de Desarrollo Tecnológico – DDT, Centro Universitario Regional del Este (CURE), Universidad de la República, Ruta 9 y Ruta 15, CP 27000, Rocha, Uruguay; [84]College of Southern Nevada; [85]University of Guelph; [86]Future Water Research Institute, Faculty of Engineering & Built Environment, University of Cape Town, Cape Town, South Africa.

*Corresponding author
‡ Unaffiliated

This file includes:

- **Table of Contents for the Supplemental Datasets**
- **List of abbreviations**
- **Quality Control and Assurance**
- **Statistical analysis of socioeconomic variables and API concentrations**
- **Image 1.** Miniaturised sampling kit used for all sampling campaigns in this global study (1)
- **Figure S1.** Distribution of cumulative concentrations of eight therapeutic classes of pharmaceuticals across Africa (n=26 sampling campaigns), Asia (n=26 sampling campaigns), Europe (n=44 sampling campaigns), North America (n=17 sampling campaigns), Oceania (n=8 sampling campaigns) and South America (n=11 sampling campaigns). Datapoints are only plotted for therapeutic classes where at least one representative API was found above the limit of quantification (LOQ). Concentrations are presented on a Log scale, hence, the plotted distributions begin at a percentile representing API concentrations >1ng/L.
- **Figure S2.** Mean composition by therapeutic class of the cumulative pharmaceutical concentration across all low-to-middle and high-income countries with significant differences (p defined as <0.05 in one-way ANOVA tests) between composition of respective therapeutic classes marked by (*). Cumulative pharmaceutical concentration is provided in brackets below the respective pie charts. Note: the Antarctic samples were excluded from this analysis due to a lack of appropriate GNI- index data.
- **Figure S3.** Analysis of sampling campaign cumulative API concentration deviation from respective national mean concentrations across 51 sampling campaigns (n=366 sampling sites) representing 6 continents. The green zone represents acceptable deviation of 1 log unit based on recent spatially explicit pharmaceutical exposure models in rivers.
- **Supplemental References**
- **Acknowledgments**

Table of Contents for the Supplemental Datasets:

Dataset S1. List of the active pharmaceutical ingredients (APIs) organised by therapeutic class which were monitored in this work

Dataset S2. Details of the sampling sites included in this project as reported by project participants
Dataset S3. Overall descriptive statistics on a continental scale for all monitored APIs
Dataset S4. Database of pharmaceutical concentrations at all the sampling locations monitored in this project
Dataset S5. Detection frequencies (%) for contaminants detected across all monitoring campaigns
Dataset S6. Socioeconomic indicators across all sampling campaigns and results of statistical analysis between cumulative API concentrations and income classifications
Dataset S7. Concentrations of key therapeutic classes of pharmaceutical contaminants observed across respective sampling campaigns
Dataset S8. Deviations of total API concentrations determined for sampling campaigns with those of the national mean total API concentration in respective countries
Dataset S9. API concentrations and socioeconomic data used in DISTLM and dbRDA analysis
Dataset S10. The sequential test that presents the progressive combination of the five selected socioeconomic factors, beginning with the most significant factor
Dataset S11. Multicollinearity table that presents the related socioeconomic factors of the five most significant factors
Dataset S12. Toxicity endpoints for the studied pharmaceuticals derived from the literature

Note: Excel documents containing the Supplemental datasets accompanies this material.

List of Abbreviations:

AMR - Antimicrobial Resistance
APIs - Active Pharmaceutical Ingredients
BMI - Body Mass Index
CEC- Critical Environmental Concentration
DALYs - Disability-Adjusted Life Years
dbRDA - Distance-based redundancy analysis
DISTLM - Distance Based Linear Modelling
GDP - Gross Domestic Product
GNI - Gross National Income
LOD- Limit of Detection
LOQ - Limit of Quantification
ND - Not Detected
PNEC - Predicted No Effect Concentration
PPP - Purchasing Power Parity
USD - United States Dollar

Quality control and assurance:

Although the sample collection protocol was previously cross-laboratory validated on an international-level with the United States Geological Survey (1), field blanks provided quality control over potential field-derived interference for 35% of the sampling campaigns (n=47 field blanks). Field blanks consisted of liquid chromatography tandem mass spectrometry (LCMS)-grade analytical water subjected to the same collection methods as the environmental samples (1). The number of field blanks collected in this work were similar or greater than other large-scale monitoring campaigns [e.g., 2, 3]. During analytical runs, a QC spiked sample followed by an instrument blank was run after every 10 injections to ensure accuracy throughout analysis. Spiked QC samples consisted of Liquid Chromatography/ Mass Spectrometry-grade ultrapure water fortified with all target APIs and internal standards at 400 ng/L each (80 ng/L of internal standards) and instrument blanks were LCMS-grade water spiked with internal standards only. Prior to each analytical run, the chromatography column was eqilibrated with a series of 20 injections consisting of an equal composite of all samples included in the upcoming run. Sampling materials and methods were the same across all sampling campaigns

and analysis of the samples occurred using one method and in one laboratory. Furthermore, an extensive analysis was conducted (1) to ensure no significant sample loss occurred during shipment under various environmental conditions and transit durations. The rate of sample loss due to breakage during transit was 1.7% (18 samples arrived broken and were not included in this work). No quantifiable concentration of the target APIs was identified in the field and analytical blanks. Identification of all analytes was confirmed both *in silico* via Thermo Scientific TraceFinder 4.1 Software and by visual inspection of the chromatograms. Transition ion ratio tolerance was determined for each run as the API-specific range observed over respective calibrations.

A quality control analysis was conducted to determine the effectiveness of using grab samples as a proxy of typical pharmaceutical concentrations on a national scale. Here, the deviation of the mean cumulative concentration of APIs determined in each sampling campaign of respective countries where more than one was conducted (n=51 campaigns representing 366 sampling sites across 17 countries sampled over all 4 seasons collectively) was compared to the mean national API concentration on both temporal and spatial scales (Table S8). This analysis compiled a dataset representative section (38%) of the total dataset. A difference of 1 order of magnitude from the national mean was determined acceptable based on that used recently by long term and catchment-wide modelling of pharmaceutical concentration evaluations (e.g., 4). Analysis revealed that only 5.9% of the calculated cumulative API concentrations (i.e., 3 of 51 tested) deviated from their respective national mean values by more than one order of magnitude (Fig S3). This indicates that grab samples can be justifiably used to represent typical pharmaceutical concentrations on a national and temporal scale when collected using the criteria set out in this work.

Statistical analysis of socioeconomic variables and API concentrations:

To determine the relationship between specific socioeconomic variables and API pollution, distance-based linear modelling (DISTLM) and distance-based redundancy analysis (dbRDA) were used. Relevant data of the socio-economic factors were extracted from four different open databases, including The CIA World Factbook (5), The FAO Aquastat (6), The World Bank Open Dataset (7) and The World Health Organisation Global Health Observatory (8). As complete datasets of socioeconomic indicators were not available for some countries, the initial analysis started with fewer countries but a maximum number of socioeconomic factors. The analysis was then repeated with fewer socioeconomic factors that were identified to be significant in the initial analysis and an increased number of countries. In total, 31 indicators and 84 countries were evaluated (Table S9-11).

Prior to the DISTLM analysis, cumulative concentrations of each of the eight classes of pharmaceuticals collected from multiple rivers in the same country were first averaged. The average pharmaceutical concentrations were fourth-root-transformed to minimize the influence of extreme values and formulated on an Euclidean distance resemblance. Socioeconomic factors were log(x+1)-transformed and standardized by their individual mean and standard deviation. During the analysis, socioeconomic factors were used as independent variables, while pharmaceutical concentrations were used as dependent variables. Different combinations of the socioeconomic factors were screened using Primer with PERMANOVA+ (v7.0.17, Primer-e). The best combination of socioeconomic factors with the smallest modified Akaike Information Criterion (AICc) and largest r2 was identified using the embedded “BEST” function in DISTLM. A sequential test was also performed with the “FORWARD” function to illustrate the sequential combination of the identified factors, starting from the most significant. A dbRDA diagram was also plotted using the identified factors.

Supplemental Image:

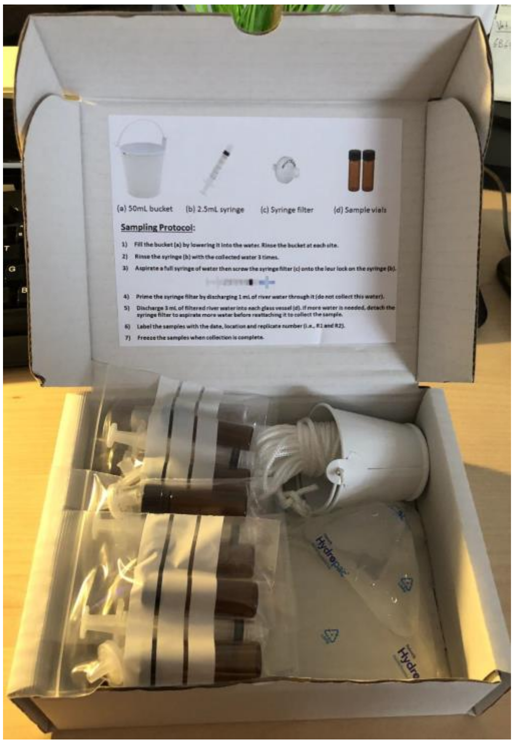

Image S1. Miniaturised sampling kit used for all sampling campaigns in this global study (1)

Supplemental Figures:

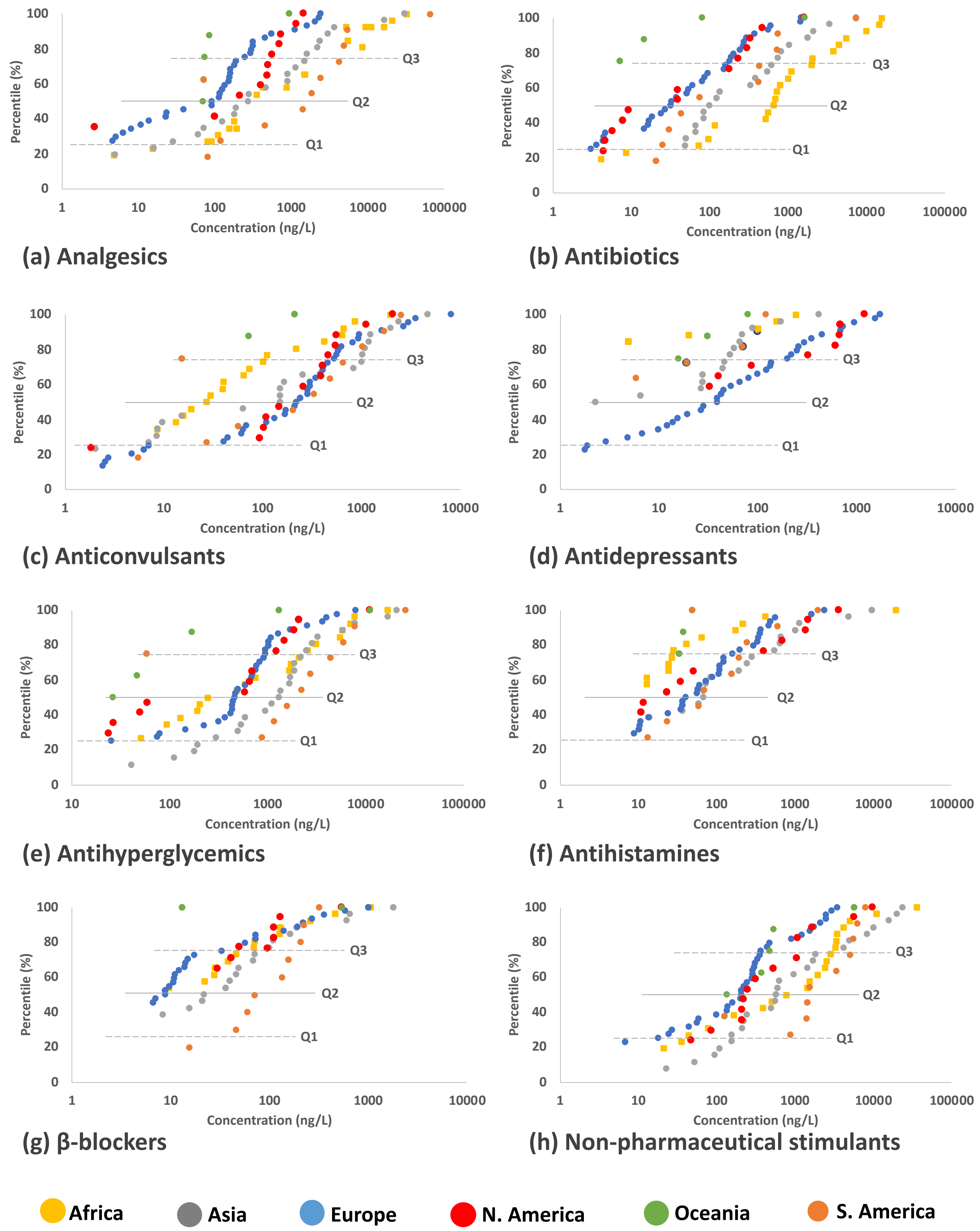

Figure S1. Distribution of cumulative concentrations of eight therapeutic classes of pharmaceuticals across Africa (n=26 sampling campaigns), Asia (n=26 sampling campaigns), Europe (n=44 sampling campaigns), North America (n=17 sampling campaigns), Oceania (n=8 sampling campaigns) and South America (n=11 sampling campaigns). Datapoints are only plotted for therapeutic classes where at least one representative API was found above the limit of quantification (LOQ). Concentrations are

presented on a Log scale, hence, the plotted distributions begin at a percentile representing API concentrations >1ng/L.

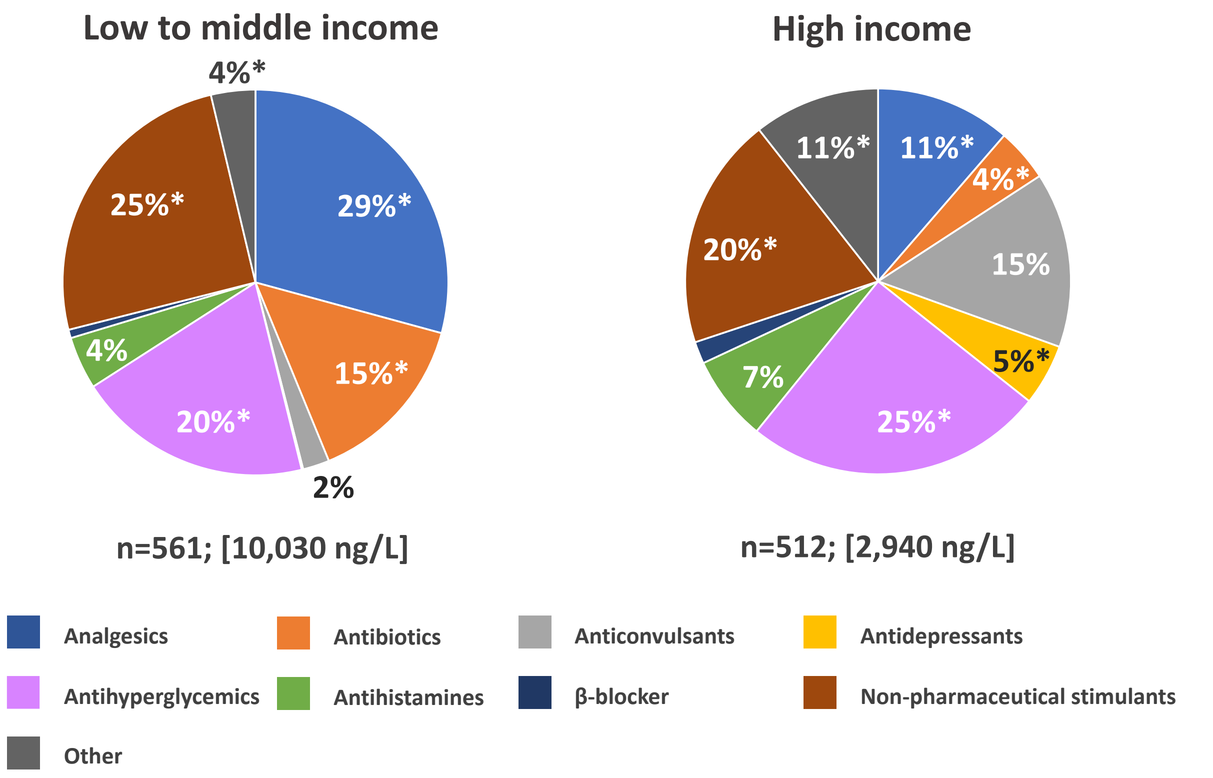

Figure S2. Mean composition by therapeutic class of the cumulative pharmaceutical concentration across all low-to-middle and high-income countries with significant differences (p defined as <0.05 in one-way ANOVA test followed by Tukey's Post Hoc test) between composition of respective therapeutic classes marked by (*). Mean cumulative pharmaceutical concentration is provided in brackets below the respective pie charts. Note: the Antarctic samples were excluded from this analysis due to a lack of appropriate Gross National Income- index data.

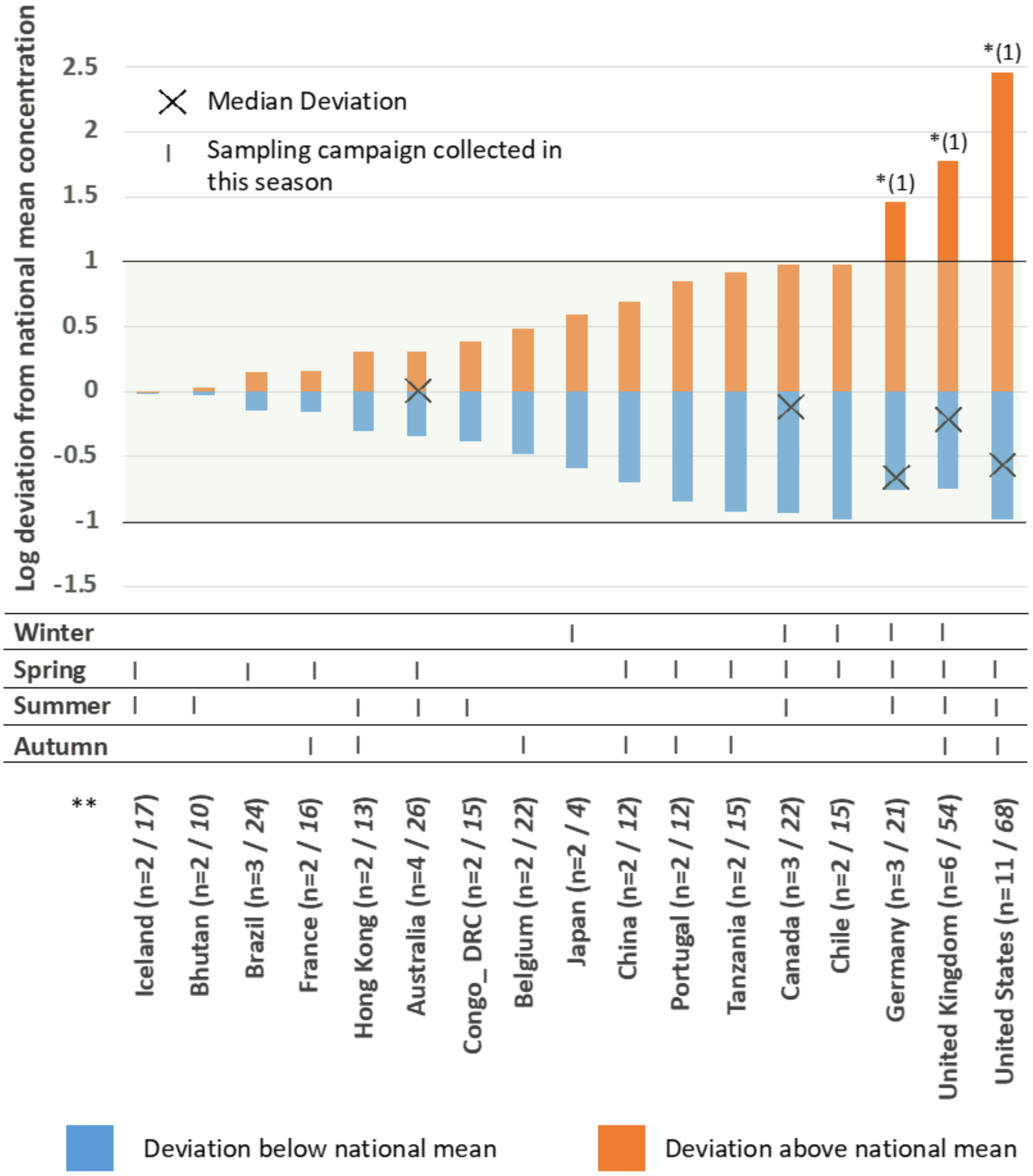

*Number of sampling campaigns (of 51 total in this analysis) deviating from the national mean concentration by > 1 order of magnitude
** 'n-value' refers to the (number of sampling campaigns conducted in respective countries / *number of sampling sites nation wide*)

Figure S3. Analysis of sampling campaign mean cumulative pharmaceutical concentration deviation from respective national mean concentrations across 51 sampling campaigns (n=366 sampling sites) representing 6 continents. The green zone represents acceptable deviation of 1 log unit.

Supplemental References:

1. Wilkinson, J.L., Boxall, A. and Kolpin, D.W., 2019. A novel method to characterise levels of pharmaceutical pollution in large-scale aquatic monitoring campaigns. *Applied Sciences*, *9*(7), p.1368.
2. Kolpin, D.W., Furlong, E.T., Meyer, M.T., Thurman, E.M., Zaugg, S.D., Barber, L.B. and Buxton, H.T., 2002. Pharmaceuticals, hormones, and other organic wastewater contaminants in US streams, 1999− 2000: A national reconnaissance. *Environmental science & technology*, *36*(6), pp.1202-1211.
3. Barnes, K.K., Kolpin, D.W., Furlong, E.T., Zaugg, S.D., Meyer, M.T. and Barber, L.B., 2008. A national reconnaissance of pharmaceuticals and other organic wastewater contaminants in the United States—I) Groundwater. *Science of the total environment*, *402*(2-3), pp.192-200.
4. Oldenkamp, R., Hoeks, S., Čengić, M., Barbarossa, V., Burns, E.E., Boxall, A.B. and Ragas, A.M., 2018. A high-resolution spatial model to predict exposure to pharmaceuticals in European surface waters: EPiE. *Environmental science & technology*, 52(21), pp.12494-12503.
5. CIA, 2021. The World Factbook, *United States Central Intelligence Agency (CIA)*, [online], last access 7 May 2021, available at: <https://www.cia.gov/the-world-factbook/>.
6. FAO, 2021. AQUASTAT Global Information System on Water and Agriculture, *Food and Agriculture Organization of the United Nations (FAO)*, [online], last accessed 7 May 2021, available at: <http://www.fao.org/aquastat/statistics/query/index.html>.
7. World Bank, 2021. DataBank: World Development Indicators. World Bank Group, [online] available at: <https://databank.worldbank.org/home>, last accessed: 9 May 2021.
8. WHO, 2021. The Global Health Observatory, *World Health Organization (WHO)*, [online], last accessed 9 May 2021, available at: <https://www.who.int/data/gho/data/indicators>.
9. Tell, J., Caldwell, D.J., Häner, A., Hellstern, J., Hoeger, B., Journel, R., Mastrocco, F., Ryan, J.J., Snape, J., Straub, J.O. and Vestel, J., 2019. Science-based targets for antibiotics in receiving waters from pharmaceutical manufacturing operations. *Integrated environmental assessment and management*, 15(3), pp.312-319.

Acknowledgments:

The authors would like to thank the Centre of Excellence in Mass Spectrometry at the University of York where the mass spectrometer that performed this work is located.

The project was partly supported by the Medical Research Council (Project: MR/R014876/1), the British Council Institutional Links STREAM program (Project Number 277947262), the Instituto Antartico Chileno Regular Funding Projects (REF: INACH_RT_12_17), ANID PIA Anillo INACH ACT192057 and ANID FONDECYT 1210946. The Slovenian water sample collection was financed by the Slovenian Research Agency (ARRS), Research Programme P4-0092.

The authors would like to thank Amanda Wong and Katherine Wong for their help by collecting water samples in Calgary, Canada as well as Prof. M. M. Pathmalal for help in the collection of samples in Sri Lanka.

Any use of trade, firm, or product names is for descriptive purposes only and does not imply endorsement by the U.S. Government.
